# Exploring the impact of teacher expectations on student behavior and academic performance using cognitive behavioral therapy

**DOI:** 10.3389/fpsyg.2026.1827270

**Published:** 2026-08-19

**Authors:** Yehui Jin, Qiang Zhou, XinJuan Li

**Affiliations:** 1 School of Mechanical and Vehicle Engineering, Nanchang Institute of Science and Technology, Nanchang, Jiangxi, China; 2 Nanchang Industrial Engineering School, Nanchang, Jiangxi, China; 3 School of Education, Nanchang Institute of Science and Technology, Nanchang, Jiangxi, China

**Keywords:** academic performance, cognitive behavioral therapy, negative expectations, student behavior, teacher expectations

## Abstract

This study evaluated the effectiveness of using a cognitive-behavioral therapy based teacher training program to change teachers’ expectations of students and improve student behavior and academic performance. This paper designs and uses a quasi experimental approach with a non equivalent control group for pre-test/post test. The participants consist of 115 third grade teachers and 356 third grade students from five primary schools. Teachers identified as having low expectations received a 12 week manual CBT training program, while those identified as having high expectations were used as the control group. This study used the Teacher Expectations Scale to measure teacher expectations, the Student Behavior Observation Scale to measure changes in student behavior, and standardized Chinese and math tests to measure improvements in student academic performance. The results showed that after implementing the intervention, the teacher’s expected score significantly increased from the baseline original ratio to a higher score (after the intervention) through statistical analysis (*t* = 4.15, *p* < 0.001; Cohen’s *d* = 0.72). In addition, the number of students who scored over 80 points in academic tests increased during the five testing processes (40.40% in the pre-test and 76.20% in the post test); also, compared with baseline measurements, the positive behavior rate of students taught by teachers who received intervention increased, while the negative behavior rate decreased.

## 1 Introduction

In school education, teacher expectations are among the most influential psychological factors affecting student academic outcomes, operating through both direct instructional effects and indirect self-fulfilling prophecy mechanisms. Teacher expectations are the beliefs and expectations teachers hold about student performance, which can influence students’ beliefs and self-evaluations and thus affect their behavior and academic performance. However, teacher expectations are not always objective and can sometimes be influenced by subjective factors. Therefore, exploring the impact of teacher expectations on student behavior and academic performance can help comprehensively understand their role, promote teacher professional development, and improve the quality of school education.

Teachers’ expectations of students have a significant impact. Teacher expectations can not only affect students’ self-esteem and confidence, but also influence their evaluation of their own abilities. Papageorge Nicholas W. suggested that teacher expectations may affect students’ completion of their university studies. He formalized this idea using a measurement-error model, estimating that the elasticity of the college graduation rate with respect to teacher expectations is 0.12. If there were a return of optimism or an optimistic demographic gap in society, this expectation would have been counterproductive (Papageorge Nicholas et al., 2020). More and more evidence suggests that teacher race and ethnic matching, as well as classroom diversity, are beneficial for students’ academic and socioemotional development. However, little was known about whether the impact of teacher race and ethnic matching varies across levels of classroom diversity.

Rasheed Damira S. tested the impact of paired teacher reports on child outcomes across samples of teachers and children of different races/ethnicities, and used a multilevel model to account for classroom diversity. It was found that the racial/ethnic matching between teachers and children is associated with higher levels of children’s participation in learning, motivation, social skills, and lower absenteeism rates (Rasheed et al., 2020). Bardach Lisa aimed to systematically describe the psychological characteristics of teachers, such as motivation and personality, and their role in key outcomes such as teacher efficacy, teacher happiness, retention, and positive interpersonal relationships with multiple stakeholders. Bardach and colleagues’ integrative review documented correlational evidence linking teacher psychological characteristics to effectiveness and wellbeing, yet its primary contribution was descriptive rather than prescriptive, as it did not examine whether these characteristics are amenable to modification through structured interventions. The present study addresses this prescriptive gap by testing whether one specific psychological characteristic—expectation patterns—can be systematically altered through CBT techniques. In terms of practical recommendations, the focus should be on teacher selection and the design of future professional development activities, as these areas particularly benefit from a profound understanding of the different characteristics of psychological teachers in promoting adaptive outcomes (Bardach et al., 2022). Teachers are crucial to maintaining a safe, efficient, and effective learning environment by cultivating student engagement and limiting destructive behavior. Although experts generally agree on the importance of cultivating classroom and behavioral management skills, many teacher training programs do not require specialized courses or experience to develop the skills required for teachers to complete their degrees. Stevenson Nathan A. described the observed disconnect between the current requirements of teacher preparation plans and the essence of supporting student behavior, which, in turn, leads to teacher turnover (Stevenson et al., 2020). Stevenson and colleagues’ critique of teacher preparation programs highlights a structural problem in teacher education. Still, their analysis does not offer a solution for equipping in-service teachers with the cognitive skills needed to maintain high expectations. The CBT protocol proposed in the present study represents one such solution, as it provides practicing teachers with transferable cognitive tools that can be applied across diverse classroom situations without requiring systemic changes to teacher preparation curricula. The use of cognitive-behavioral therapy to address teacher expectations can help teachers adopt a more positive attitude toward expectations and promote student self-affirmation and development.

Cognitive-behavioral therapy can help individuals change unhealthy thinking and behavioral patterns. The destructive behavior of middle school students is related to negative outcomes, including low grades, dropout, and teacher burnout. The purpose of Zoromski and Allison was to evaluate the rates of disruptive and task-based student behavior in high school classrooms, including group/peer assignments, group teaching, and overall group teaching, as well as the observed (appropriate and inappropriate) command and marked/unmarked praise response opportunities. The results indicated significant differences among teachers in their use of trust strategies, their perceptions of trust strategy effectiveness, and the frequency of destructive student behavior. Zoromski and colleagues documented substantial variability in teachers’ classroom management practices and observed that this variability was associated with student behavioral outcomes. The cross-sectional design of their study, however, left unanswered whether training teachers in specific cognitive-behavioral techniques would reduce this variability by raising the performance of lower-functioning teachers toward the level of their higher-functioning colleagues. The present intervention study directly tests this equalization hypothesis. It is worth noting that the strongest correlation is between appropriate responses to destructive behavior and the overall ratio of destructive to task behavior (Zoromski et al., 2021). Supporting social–emotional learning programs is a way to enhance adolescent psychological resilience and promote positive mental health. LaBelle Brittany reviewed the literature on using this program to address students’ psychological health needs in school environments. Several studies targeting different populations have found that this project has indeed improved personality development, reduced psychological problem behaviors among students, and enhanced their adaptability. Necessary connections have been established between social and emotional learning projects that have already been created and developed, and these projects are clearly linked to cultivating students’ adaptability (Brittany, 2019). Asanjarani Faramarz used an experimental design to examine the effects of cognitive-behavioral therapy on math anxiety and math self-concept among elementary school students. Thirty students with high math anxiety and low math self-concept were selected from 142 elementary school students in Arak, Iran. Participants were randomly assigned to a control group or a treatment group, and math anxiety and math self-concept were measured using a math anxiety assessment scale. The research results showed that participants in the treatment group had lower mathematical anxiety and higher mathematical learning ability after participating in cognitive behavioral therapy interventions (Faramarz and Zarebahramabadi, 2021). The cognitive intelligence of young children has not been fully developed in schools and needs to be highly valued. Rosyadi B. R. aimed to analyze and understand the impact of teacher expectations on children’s cognitive intelligence, using a qualitative case study method. The results indicated that teacher expectations were planned and systematic in developing children’s cognitive intelligence, and research suggested the importance of implementing high-expectation education for teachers (Rosyadi et al., 2021). The application of cognitive-behavioral therapy can help teachers better understand student behavior and learning disabilities and design corresponding educational interventions.

Research has thoroughly examined teacher expectations and their effects on students, including factors around developing expectations and the differences in behaviors of teachers with high and low expectations; but there still continues to be an area of research needing further elaboration. While many researchers have shown relationships between teacher expectations and student achievement, they have primarily provided descriptive information about either how teacher expectations were formed and ways to offer positive experiences for students in the classroom. For example, the existing literature on teacher training has been focused largely on educating teachers regarding the negative implications of having low expectations for students, and creating structured classroom activities whereby teachers can provide positive experiences for students. These efforts have yielded inconsistent findings, and have failed to assist students in removing their negative perceptions and their low expectations. Cognitive-behavioral therapy provides a well-founded theoretical and empirical basis from which teachers can begin to understand the negative perceptions and low expectations that often result from specific cognitive errors, including overgeneralization of isolated student failures, selective abstraction of negative behavior evens that occur from their students, and or dichotomous thinking (i.e., perceiving students as either capable or incapable). The core premise of cognitive-behavioral therapy suggests that maladaptive cognitive-error patterns can be identified, disputing the pattern through systematic processes, and restructuring through systematic processes will assist with reducing the teacher’s cognitive-error patterns that contribute to their teacher expectations. The reason to justify applying CBT with regard to altering teacher expectations is that this approach addresses expectations through cognitive precedents instead of only through addressing the visible behavior associated with an expectation. The following conceptual framework will shape current study: When the teacher implements these behavioral changes based upon heightened expectation, then the student recognizes the behavioral changes as indicators of teacher confidence; therefore through an increase in confidence by the teacher, the student develops higher levels of self-efficacy, provides greater academic effort, and acts positively in the classroom; as a result of all of these student responses. Students will achieve measurable increases in academic performance.

In order to address the research gap identified above and test the proposed conceptual framework, an empirical investigation was designed to examine how a CBT-based intervention would affect teacher expectations and student outcomes. The remainder of the document will present the theoretical framework underlying the intervention, as well as the methodology used to conduct the empirical investigation, and will then review the results of the investigation.

## 2 Methodology

### 2.1 Research design and participant selection

The study utilized a quasi-experimental pre-test/post-test design with a non-equivalent comparison group.

The sample consisted of 356 third-grade students and 115 teachers from 5 public primary schools located in a mid-sized city in eastern China. A multi-stage stratified random sampling procedure was used to recruit participants. All public primary schools located within the target city were stratified according to their total student enrolment, with three strata established: tier 1 (>800 students), tier 2 (500–799 students), and tier 3 (<500 students). Two schools were randomly selected from both tier 1 and tier 2, and one school was randomly selected from tier 3, for a total of 5 schools participating in this study. Each selected school had a complete list of its third-grade classrooms, and two classrooms were randomly selected from each school using a random number generator, for a total of 10 classrooms. All students in the randomly selected classrooms were invited to participate; the only exclusion criteria were students with developmental disabilities or students who had missed more than 15 days of school during the academic year.

### 2.2 Demographic characteristics

Participation in the study was composed of 182 male and 174 female students having a mean age of 9.2 years (SD = 0.5). Socioeconomic status was assessed based on two measures including maternal educational attainment and monthly family income per capita. Educational attainment of parents was as follows: 67 parents had earned at least a bachelor’s degree; 143 parents had earned a high school diploma or an associate degree; and 146 parents only had completed some middle school. The distribution of family monthly income was: (1) below 3,000 RMB, (2) between 3,000 RMB and 6,000 RMB, and (3) above 6,000 RMB. Academic performance prior to the study was measured based on each participant’s school record from the semester immediately prior to this research project. Mean scores for each participant when measured by subject area were 74.6 (SD = 11.2) for Chinese, and 71.8 (SD = 12.4) for math. 14.6% of students had an academic performance score of less than 60, 51.7% between 60 and 80, and 33.7% above 80. Of the 115 teachers in the sample, 89 of the teachers were females, and 26 were males. The mean number of years teachers had taught prior to this research project was 11.4 years (SD = 6.7). Teacher demographics were collected using a teacher self-administered demographic questionnaire that measured various demographic characteristics and included teacher’s ages. Teacher ages ranged from 24 to 54 years and had a mean age of 37.8 years (SD = 8.2). 32 teachers (27.8%) had a master’s degree; 79 teachers (68.7%) had a bachelor’s degree; 4 teachers (3.5%) had an associate degree. The teaching experiences were separated among the participants as follows: Of the participants, 28 teachers (24.3%) provided 1–5 years of experience; 31 (27.0%) provided 6–10 years of experience; 35 provided 11–15 years of experience (30.4%); and 21 (18.3%) provided more than 15 years of experience. The 62 of 115 (53.9%) teachers specializing in Chinese language and literature; 42 of 115 (36.5%) in mathematics; and 11 of 115 (9.6%) in other disciplines/fields. All of the teachers that participated in the study had completed accredited teacher education programs and worked at the same school for an average of 8.6 years (SD = 5.4 years). Student participants were recruited with written consent from their parents through a two-step process. First, the parents were provided with a detailed information letter outlining the research study’s purpose, the nature of classroom observations, the nature of academic testing, and the right to withdraw consent at any time without penalty. After receiving written parental consent, the parent received a second letter outlining what data would be collected about their child and how that data would be secured for their child’s privacy. The children were given a verbal description of the study by age and were asked to indicate whether they would like to participate with a child-friendly assent form. The following protections would be applied to the students: to ensure no student data are able to be identified, all identifiers are removed from their records and each student is given a unique participant ID to link them to their de-identified record; and that all identifiable information is maintained by the principal investigator in a locked office and is only accessed by the principal investigator. Consent forms will be kept separate from data, and the research protocol was given ethical approval from the Institutional Review Board; as part of that approval, the IRB reviewed all of the protocols for obtaining parent consent and child assent, data protection protocols and how the researcher would handle any adverse events.

### 2.3 Intervention protocol and implementation

The research occurred over 3 distinct phases. In the first phase, baseline data were collected for teachers’ expectation levels, student behavior observations, and academic performance records. The second phase consisted of the intervention, during which teachers identified as having low expectations (*n* = 78 teachers with 254 students) received a CBT-based intervention. Teachers who were identified as having high expectations provided a natural comparison group for this study (*n* = 37 teachers with 102 students). Phase three of the research included data collection after the intervention was completed, during the final week of the 12-week period.

The protocol for low-expectation teachers consists of a manualized CBT program delivered in 12 weekly Sessions of 90 min each. Four intervention-specific CBT techniques were selected based on their theoretical relevance to changing expectation. The main intervention technique was cognitive restructuring: It followed a 5-step process using the following steps: identification of automatic negative thoughts regarding individual students, examining evidence for and against the automatic negative thoughts identified, development of alternative explanations that account for individual differences among students, evaluation of the alternative explanations against student data collected, and the use of the most evidence-based appraisal of the student in question. The second intervention technique was behavioral experimentation: It involved a 4-step process as follows: identify a specific classroom submissive behavior or situation in which the teacher usually has negative expectations of students, predict how the students would behave differently when given a modified instructional approach, implement the new instructional approach and collect data on the students’ actual responses by observing their behavior, and compare the students’ actual responses to the teacher’s predictions in order to generate disconfirming evidence. The third technique used in the intervention was problem-solving training: Teachers were assisted in solving specific problems in the classroom which trigger negative expectations such as noncompliance, academic failure, and behavioral disruption. The fourth technique was self-monitoring: Teachers were required to keep daily logs of their thoughts about students, their emotional responses to student-related thoughts, and their behavioral reactions to students’ behavior relative to the teachers’ thoughts. The program was done as a group intervention, which had a minimum of six and maximum of eight teachers per group. Each group was co-facilitated by two licensed clinical psychologists. The group format allowed for modelling of new behaviors and for gaining support from the peers in the group. The manual provided all the necessary components for delivering the program, i.e., detailed outline and timing of each activity and the introduction scripts for each technique. Immediately after completion of each session, co-facilitators completed a checklist for adherence to the manual to rate how well each component of the manual was delivered at each session. There was an adherence rate of 93.7% (*N* = 419) for the manual across all sessions. All teachers participated as low expectant teachers, meaning none of the students received CBT sessions from their individual teacher. During the entire intervention period, students did not receive any type of psychological intervention and only received the modified instructional behaviors from their teachers who received the intervention. Each session had three distinct segments: cognitive assessment and emotion monitoring—20 min; core intervention—50 min; action planning and feedback—20 min. The content developed for each session was developed in a sequential manner to enable cognitive and behavioral skills to be learned gradually and sequentially. A detailed summary of the progression of this 12-week intervention is found in Table 1.

**Table 1:** Twelve-week cognitive behavioral therapy intervention protocol.

Session	Core objective	Intervention content
Week 1	Therapeutic alliance	Introducing the cognitive behavioral model of teacher expectations
Week 2	Thought identification	Identifying automatic negative thoughts during classroom interactions
Week 3	Distortion recognition	Analyzing cognitive distortions encompassing overgeneralization and dichotomous thinking
Week 4	Cognitive restructuring	Challenging negative appraisals using objective behavioral evidence
Week 5	Alternative appraisals	Generating balanced and objective interpretations of student behaviors
Week 6	Experiment design	Formulating behavioral predictions under modified teaching conditions
Week 7	Initial implementation	Executing structured behavioral experiments in the classroom environment
Week 8	Outcome evaluation	Reviewing discrepancies between predictions and actual student responses
Week 9	Advanced application	Implementing targeted experiments for challenging student interactions
Week 10	Skill reinforcement	Maintaining expectation gains amidst routine educational setbacks
Week 11	Relapse prevention	Formulating personalized cognitive maintenance plans
Week 12	Protocol consolidation	Conducting comprehensive review and therapeutic relationship termination

The cognitive restructuring and behavioral experiments were utilized as primary methods of structuring the intervention by the two licensed clinical psychologists delivering the interventions in this study, both of whom were previously trained to provide school-based focus. Each of the facilitators had earned their doctorate degree in clinical psychology from an accredited university and had completed a minimum of 2,000 h of supervised cognitive-behavioral therapy (CBT) prior to beginning the study. Each of the facilitators also had at least three years of delivering CBT in an educational setting and had received two days of training specifically for implementing the present intervention protocol. The training included didactic information on the theoretical basis of teacher expectations, role-played demonstrations of each of the intervention sessions, and actually practicing how to use the fidelity rating instruments (FRIs). Facilitators of the intervention participated in weekly group supervision meetings with a supervising clinical psychologist, who had 20 years of experience in CBT, during which they reviewed and discussed difficult cases and discussed deviations from protocol implementations. Through the use of session recordings and weekly supervisor meetings, treatment fidelity was monitored. Three complementary procedures were used to evaluate fidelity of implementation. Each session of an intervention was recorded in audio with participant consent; 25% of the recorded sessions were selected at random for independent evaluation by two trained research assistant not involved in delivering the intervention. The evaluation was completed using a standardized Fidelity Checklist consisting of 12 items related to the extent to which the session adhered to the prescribed contents (6 items), quality of therapeutic delivery (3 items), and participant engagement (3 items), with each item being assigned a score on a scale of 1–3. The inter-rater reliability of the Fidelity Checklist was 0.91. A self-report Session Completion Log (included in the facilitator’s protocol) was completed by the facilitator at the end of each session. This log contained information about any additional components that were accomplished; what modifications were made to the protocol; and any factors influencing deviation from the protocol. Structured fidelity review was a component of each weekly supervisory meeting where audio excerpts of recorded sessions were played for the facilitator to evaluate how he/she met fidelity standards. Across all of the reviewed sessions, the total fidelity rate was 92.3% with individual session fidelity percentages ranging from 86.7–96.5%. There were no sessions excluded from analysis based upon concerns related to the fidelity of the session.

### 2.4 Measurement instruments

#### 2.4.1 Teacher expectation scale

The Teacher Expectation Scale (TES) is a 24-item self-reported tool for measuring teacher expectation for students’ academic achievement, behavioral conduct, social competence and future potential. The TES was validated using multiple methods. A panel of five educational psychology experts was used to determine content validity. Construct validity was determined through confirmatory factor analyses, which verified a four-factor structure. Criterion validity was assessed by examining the correlation between TES scores and observed teacher behaviors in the classroom; the correlation coefficient was *r* = 0.61, *p* < 0.001. The validation sample of 120 teachers from non-participating schools was chosen so as not to overlap with the sample used for the primary research. Each item is rated as a 5-point Likert scale, i.e., the higher the score, the greater the expectation. The TES is based on a thorough review of the existing literature on teacher expectations and adapts the Teacher Expectation Scale originally validated by Jussim and Eccles (1992). Internal consistency reliability for the current sample was 0.84 for the overall scale, and subscale alphas were between 0.76 and 0.82. Test–retest reliability over a 2-week period was examined with a subsample of 30 teachers, finding it to be *r* = 0.79.

#### 2.4.2 Student behavior observation checklist

The Student Behavior Observation Checklist (SBOC) was created based on systematic observational protocols derived from a Classroom Behavior Observation System developed by Caldarella et al. (2020). Positive behavior observation criteria included active participation, assignment completion, cooperation with peers, following rules, asking questions of others, helping others, volunteering for responses, and maintaining sustained attention to tasks. Negative behavioral categories included avoiding tasks, being late, cheating, and having low self-esteem. In addition to being observed, all students received two independent 30-min classroom observations by trained research assistants twice per week. Teachers did not provide ratings of their own students in an effort to reduce expectancy bias and students were not allowed to complete self-report measures regarding their behavior in order to reduce social desirability bias. Research assistants were required to participate in 20 h of observation protocol training, achieving at least 85% agreement with previously coded master video clips prior to independent observations. A detailed observation manual for each behavior was created containing operational definitions, examples of behaviors included in the operational definitions, and exclusionary criteria. Inter-rater reliability for the present study was established through paired observations conducted by two trained research assistants across 30 separate classroom observation sessions, yielding Cohen’s κ coefficients ranging from 0.81 to 0.93 for individual items and an overall κ = 0.87. Construct validity was assessed through confirmatory factor analysis, which supported a two-factor structure (positive behaviors and negative behaviors) with fit indices of $\chi^{2}/\mathrm{df}$ = 2.14, CFI = 0.93, RMSEA = 0.06. Internal consistency was α = 0.86 for the positive behavior subscale and α = 0.79 for the negative behavior subscale.

#### 2.4.3 Academic achievement tests

Two types of standardized academic achievement tests (Chinese and mathematics) were completed at baseline and post-intervention. These tests consist of district-wide standardized tests administered by the Bureau of Education at the end of each semester; the tests were created by a panel of experienced classroom teachers and curriculum experts according to a rigorous testing process that adhered to the national curriculum content. The Chinese test was organized into three sections: reading comprehension (35%), writing (30%), and language fundamentals (35%) (e.g., vocabulary and grammar). The mathematics test was divided into four sections: number operations, estimating, measurement, geometry (35%); data analysis, and probability (20%) (25%), and problem-solving (20%). The internal reliability coefficients were *α* = 0.88 for the Chinese test and *α* = 0.86 for the mathematics test; the reliability of each test was evaluated by assessing test–retest reliability with 120 students (all of whom were not involved in the research) who were retested over a 3-week period (*r* = 0.82 for Chinese, *r* = 0.79 for mathematics). All academic tests were administered under standardized test administration conditions; only teachers who were not involved in the pilot participated as test proctors, followed standardized protocols regarding allowable materials, time limits, and seating arrangements. In addition, each student’s raw score was recorded and then converted to percent scores for data analysis.

#### 2.4.4 Student perception of teacher expectations questionnaire

The Student Perception of Teacher Expectations Questionnaire (SPTEQ) is a 16-item measure that assesses students’ perceptions of teacher expectations. This instrument was adapted from the Teacher Treatment Inventory developed by Brattesani, Weinstein, and Marshall, and modified for third grade reading level through a pilot testing process with 35 non participating students (Brattesani et al., 1984). The scale assesses four dimensions of perceived teacher expectations: academic demands (4 items), feedback frequency and quality (4 items), emotional warmth (4 items), and autonomy granting (4 items). Response options were presented on a 4-point pictorial Likert scale with emoji anchors to facilitate comprehension for young children. Confirmatory factor analysis with the present sample confirmed the four-factor structure with acceptable fit: $\chi^{2}/\mathrm{df}$ = 2.31, CFI = 0.91, RMSEA = 0.07. Internal consistency reliability for the total scale was α = 0.81, with subscale alphas ranging from 0.73 to 0.79. Test–retest reliability over a 2-week interval with a subsample of 50 students was *r* = 0.76.

### 2.5 Data collection and analytical procedures

All schools that participated in the study followed standard procedures to collect data. Baseline academic scores were taken from the school records based on the most recent standardised data. Class observations were conducted on randomly assigned days to reduce the likelihood of responding differently because of the effect of observing the class, with the observer being located in the back of the class and remaining non-participating. Post-intervention academic tests were conducted in the same manner as baseline assessments. All questionnaire data was collected in groups in the school during normal school hours, with research assistants available to assist answering questions. All data entry was checked for accuracy by two independent research assistants.

Using a conservative method for handling missing data, 12 (3.4%) of the 356 student participants were not present for at least one of the 5 academic testing occasions and were therefore dropped from the repeated-measures ANOVA. For the teacher scales, 3 teachers (3.8%) out of 78 who received the CBT intervention missed more than 2 of the intervention sessions due to schedule conflicts and were excluded from the paired-samples t-test analysis. Thus, the final analytical samples (343 students for the academic performance analysis and 75 teachers for the teacher expectation change analysis) contained no participants who had withdrawn from the study before completion. Missing data were determined to be missing completely at random based on Little’s MCAR test, $\chi^{2}$ (15) = 18.64, *p* = 0.23, and listwise deletion was applied for the affected analyses. All reported sample sizes reflect the actual number of participants with complete data for each specific analysis.

## 3 Cognitive behavioral therapy

### 3.1 Core components and techniques

**Figure 1:**
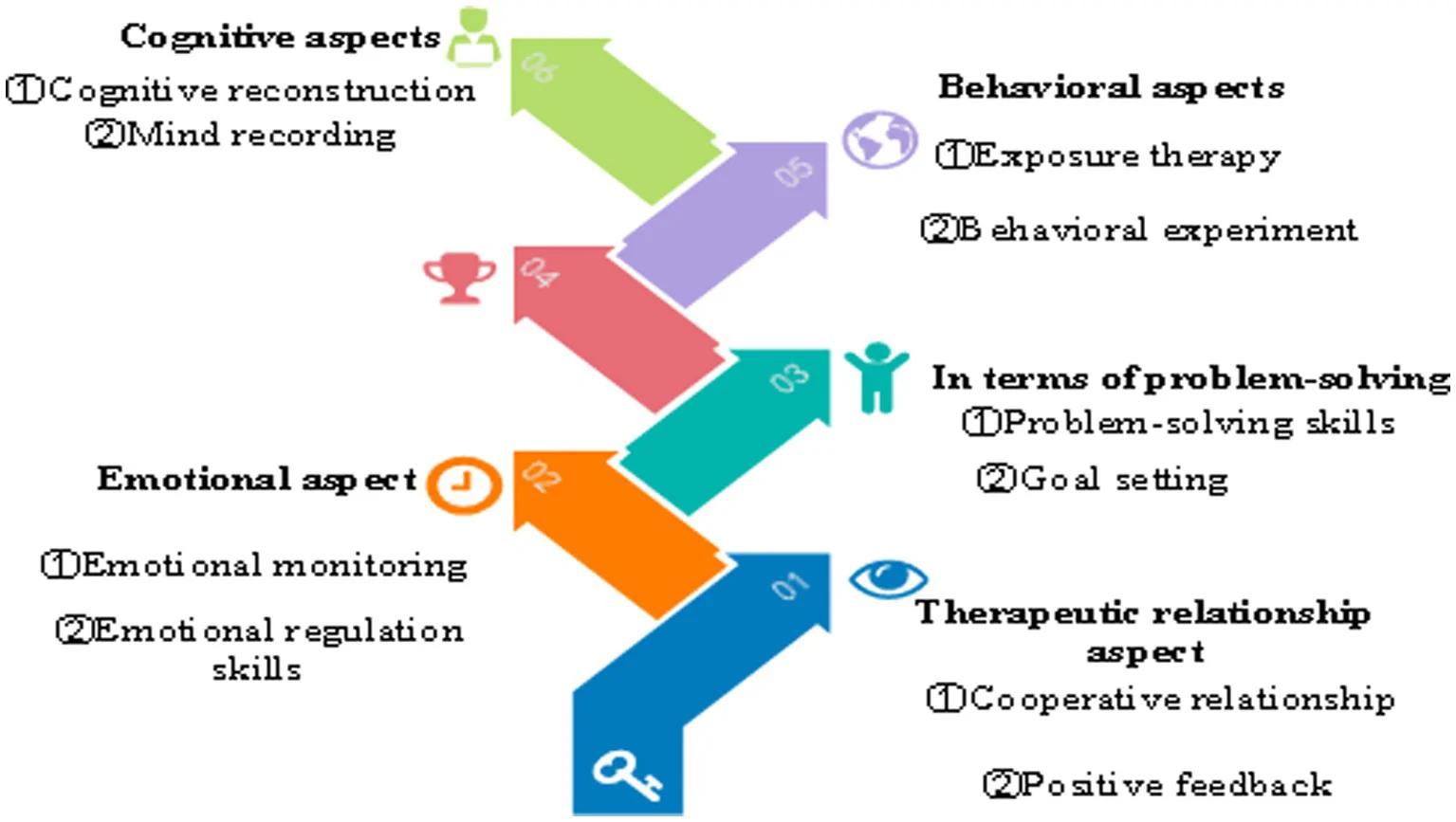
Content of cognitive-behavioral therapy.

CBT addresses maladaptive cognitive patterns and behavioral responses through structured therapeutic techniques (Ghasemi et al., 2023; Lee and Jang, 2021). The intervention targets the interconnected relationship among thoughts, emotions, and behaviors to replace distorted appraisals with balanced interpretations. The content of cognitive-behavioral therapy is shown in Figure 1.

As shown in Figure 1, the content of cognitive-behavioral therapy includes cognitive, behavioral, emotional, problem-solving, and therapeutic relationship components.

Cognitive aspects include cognitive reconstruction and thought recording, and CBT emphasizes the impact of an individual’s cognitive approach to a problem or situation on emotions and behavior. In terms of behavior, there are exposure therapy and behavioral experiments. Exposure therapy is used to treat anxiety and phobias, and individuals gradually face things they fear to reduce fear reactions. Emotional aspects consist of emotion monitoring and emotion regulation skills. By recording emotional changes, individuals can learn to control better and regulate their emotions (Situmorang, 2021; McBride and Greeson, 2023).

In terms of problem-solving, there are skills and goal-setting. CBT focuses on helping individuals develop their problem-solving skills and respond more effectively to life’s challenges. In terms of therapeutic relationships, it includes cooperative relationships and positive feedback. Positive support and feedback help individuals build confidence.

Cognitive-behavioral therapy is focused primarily on two aspects of a person’s life; the thinking part, and the behavior part. The thinking part includes techniques like cognitive reconstruction, positive responses, and self-help; while the behavior part includes techniques like behavior substitution, behavioral experimentation, and behavior planning.

### 3.2 Dual pathways of expectation modification

CBT affects how teachers expect their students to do in two main ways. It helps to change how the teacher thinks about their students’ abilities. Teachers who have low expectations for their students tend to use several types of cognitive errors. They may use overgeneralization, where they generalize about all students based on one student failure or negative behavior. They also use selective abstraction, where they tend to only remember negative behavior from a student. They may also use dichotomous thinking, where they tend to categorize students as either “can” or “cannot.” The use of CBT with a teacher, through the process of cognitive restructuring, will help to identify and change their negative automatic thoughts about a student to an empirical test based on evidence of student behavior. The cognitive restructuring process will result in lasting changes in how a teacher thinks about the ability of their students, by transforming their fixed expectations into a flexible and context-based evaluation of their students.

The second track of this process is via reinforcement of behavior. CBT utilizes behavioral experiments where educators try out different instructional strategies with students who are previously considered by that educator as less effective. The result of the experiments creates disconfirming evidence against the originally held negative expectation and provides the educator with a cognitive dissonance or cognitive conflict in which they are motivated to revise the originally held negative perceptions of students they taught. The behavioral rehearsal component of CBT also helps to increase the educators’ self-efficacy for working with diverse learners and to raise their expectations through reciprocal determinism.

### 3.3 Mediated impact on student outcomes

The effect of teachers modifying their expectation of students has a pathway to student-based modification through mediation. When a teacher has higher expectations of their students, they change their behaviors in several ways; the teacher will provide much more positive feedback, provide more difficult tasks, wait longer for students to respond, and demonstrate greater levels of warmth through eye contact and proximity to the student. Students then see these behavioral changes as telling them that the teacher has confidence in their ability to succeed, which directly contributes to the student believing in themselves and thereby increasing their academic self-concept. Once a student believes in themselves, they will have more academic effort and persistence, as well as have more mastery-oriented goals. At the same time, students will internalize the teacher’s higher expectations as self-fulfilling prophecies by raising their expectations of themselves and increasing the way in which they engage in more strategic methods of learning. The totality or cumulative effect of the cognitive and behavioral changes of both student and teacher will lead to measurable changes in both classroom behavior and standardized achievement scores.

Student attributes can influence the relationship between variations in teacher’s expectations and changes in students’ results. More sensitive to shifts in teacher’s expectations would be those students with low academic self-concept, as how teachers respond to them gives them information about where they stand academically. They lack stable internal standards by which to assess their performance. Students with high motivation to learn will be able to respond rapidly to increased levels of expectation from teachers because their current level of motivation magnifies the impact that teacher’s motivation will have on their behavior. The moderator effect of the characteristics of the individual students explains the variance of intervention effects across different groups of students.

The full conceptual framework outlining the proposed connections between the constructs of this research is developed through five sequential links. The first link assumes that CBT methods (such as cognitive restructuring and behavioral experiments) directly address teachers’ cognitive distortions relating to student capability and that these methods ultimately lead to teachers having fewer cognitive distortions. Consequently, as teachers reduce their cognitive distortions, they have higher expectations for their students; this is demonstrated through an increase in the number of positive appraisals of their students’ abilities in an academic, behavioral, and social sense. The third link suggests that an increase in teacher expectation then translates into a change in their instructional behaviors. More specifically, teachers provide their students with more positive feedback, assign more difficult tasks for their students to complete, allow for more wait time after asking their students a question, and display more non-verbal warmth toward their students. According to the fourth link, Students interpret the modification of the teacher’s behavior as a sign of confidence on the part of the teacher, thereby increasing their own self-efficacy and their academic self-concept. The fifth link states that increased perceptions of self-efficacy will also result in an increased amount of academic effort and an increased amount of persistence in finding solutions to resolvable problems as well as a stronger tendency for students to adopt outcome oriented learning goals which result in improved scores on standardized assessments. In general, the social cognitive theory of self-efficacy and the cognitive-behavioral construct of cognitive restructuring of the teacher’s perspective, represent two separate pathways within the overall framework. The model also provides insight regarding the extent to which the characteristics of students, such as baseline academic self-concept and intrinsic motivation, affect the strength of the fourth and fifth links.

### 3.4 Theoretical justification for CBT application

CBT has three theoretical bases for its use as an intervention model to alter teacher’s expectations. The first is that teacher’s expectations, maintained through automatic cognitive mechanisms that occur outside of their conscious awareness, share a common cognitive structure with the principles of CBT. For example, teacher expectations are maintained by automatic cognitive processes, such as the selective attention given to expectancy confirming information and the biased interpretation of ambiguous student behavior. CBT techniques directly address the automatic cognitive components of maintaining teacher expectations by using structured activities that bring implicit evaluations of students into conscious awareness and allow for empirical evaluations of these evaluations. A second justification for CBT is the difference between informational systems of acquiring knowledge and experiential systems of acquiring knowledge. Informationally based teacher training programs provide the obscene consequences of having low expectations (i.e., the ability to learn based on the information of others); however, informationally acquired knowledge alone will not disrupt or change automatic cognitive processes. The behavioral experiments that take place as part of CBT will produce evidence of the experiential disconfirmation of negative expectations; therefore experiential learning provides a stronger basis for the maintenance of cognitive changes. Finally, the third justification is sustainability of the intervention effects. The incorporation of relapse prevention techniques (e.g., cognitive maintenance plans and self-monitoring procedures) provides teachers with the necessary skills to continue the use of the new expectations after the formal intervention process has been completed. Together, the three justifications demonstrate that cognitive behavioral therapy (CBT) is more than just one of many potential treatment strategies; rather, CBT has a theoretical basis that matches the types of cognitive, experiential, and sustainability issues involved in modifying teacher expectations.

## 4 Impact of teacher expectations on student behavior and academic performance

### 4.1 Connotation and characteristics of teacher expectations

Teacher Expectations are the cognitive evaluations teachers make about how students will perform academically, behave, and function socially in the future. Teacher expectations are separate from teacher beliefs (generalized ideas about teaching and learning that may not pertain directly to a specific student), from teacher attitudes (affective orientations toward being a teacher), and from teacher behaviors (observable actions taken by teachers in the classroom). Teacher expectations are different from other constructs because they are related to specific students, are oriented toward the future, and may be revised based on new information about the students. The criteria for high expectations are positive evaluations of student achievement and growth, in addition to being evidenced through behavior. Some of the behavioral indicators that show high expectations are: frequently and actively communicating with students; providing individualised academic assistance; giving positive and constructive feedback; and having a belief that students are capable of meeting high standards. The criteria for low teacher expectations are negative evaluations of student achievement, and limited student growth, in addition to being evidenced through behavior. Some of the behavioral indicators that show low expectations are: infrequent/half-hearted communication with the student; lacking individualised academic assistance; providing negative/discouraging feedback; and having a belief that students cannot meet their expectations. These operational definitions guided the coding of teacher behaviors in classroom observations and the construction of the Teacher Expectation Scale items. High teacher expectations can stimulate students’ enthusiasm and confidence, and promote their academic growth and personality development; conversely, they can suppress students’ self-confidence and motivation, thereby affecting their academic performance and quality of life (Jamil et al., 2023; Dimosthenous et al., 2020).

**Figure 2:**
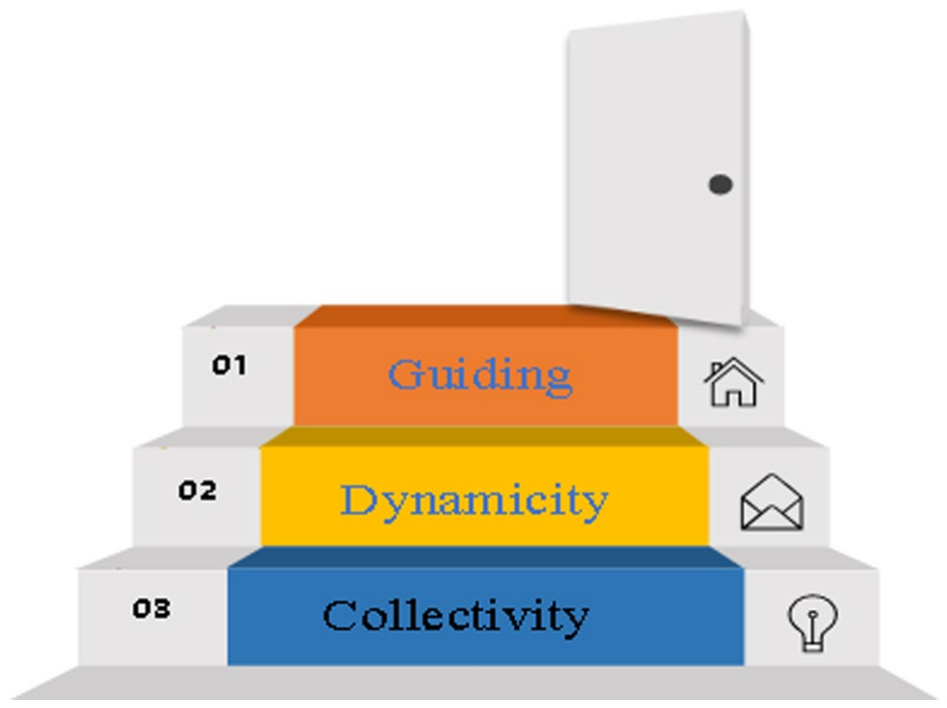
Characteristics of teacher expectations.

Teacher expectations are an educational activity conducted through language and action. Teacher expectations are not only reflected in their verbal behavior, but also in their psychology and attitude. The characteristics of teacher expectations are shown in Figure 2.

As shown in Figure 2, the characteristics of teacher expectations include directionality, dynamism, and collectivity.

Teachers’ expectations are not limited to paper grades; they are also reflected in a deep insight into students’ character, learning attitude, and potential abilities. The dynamism of teacher expectations refers to the deepening of their understanding of students and the continuous adjustment and evolution of their performance (Lei et al., 2022; Bliss and Jacobson, 2020).

Teacher expectations are collective behaviors influenced not only by individual teacher characteristics and evaluation factors, but also by school culture, policies, and the social environment. This collectivity makes teachers’ expectations not only a reflection of personal experiences but also a collective shaping and resonance across a wide range of educational contexts.

### 4.2 Impact mechanism of teacher expectations on student behavior and academic performance

Self-actualization prophecy effect: Teachers expect not only to evaluate students directly but also to stimulate students’ inner self-actualization. If teachers set high expectations for students, it would stimulate their intrinsic motivation for learning and encourage them to complete the learning tasks teachers expect more confidently (Maddocks, 2020; Li and Chu, 2021). Therefore, teachers’ positive expectations are not only an evaluation but also an incentive mechanism.

Guiding response effect: Teachers expect a guiding response effect, meaning they hope students will perform better in a particular area and actively guide and encourage them. This guiding response effect is manifested not only in the teacher’s expectations for students, but also in their guiding attitude toward student behavior and learning. By providing guided feedback, encouragement, and support, teachers can help students set clearer goals for behavior and academic standards. The guiding role of teachers in the teaching process can not only affect students’ behavior but also their learning attitudes and confidence, thereby achieving the learning goals teachers expect.

Evaluation response effect: Teacher expectations have a significant impact on students’ evaluation response effects, with the effect of praising and encouraging students with higher expectations being the most significant (Huang et al., 2020; Hossain et al., 2022). This kind of affirmative assessment is not only a recognition of student performance, but also an affirmation of individual value. With teacher motivation, students can develop a positive self-evaluation and recognize their achievements and value, thereby enhancing their confidence. This positive evaluation response can not only foster students’ positive behavior but also improve their academic performance. Teachers’ expectations of students foster a sense of value, and this virtuous cycle can not only stimulate their learning motivation but also lay a good foundation for their future development.

### 4.3 Specific impact results

The subjects of this study consists of 356 3rd grade students and 115 teachers from 5 elementary schools who were selected through the use of experimental research methods. The intended target population of the research is the basic level of education; therefore, 3rd grade students and their teachers make up the subjects of the study. Through examining a wide range of characteristics (i.e., different student and teacher backgrounds), the study will provide insight into how teacher expectations are established across different environments, as well as reveal how personality traits and prior educational experiences shape teacher expectations. By screening multiple schools and classes, the universality and variation in teacher expectations can be more comprehensively examined, providing strong support for a deeper understanding of the impact of teacher expectations on student behavior and academic performance.

The high expectations of teachers can be reflected in multiple aspects: active communication, personalized support, positive encouragement, trust, and expectations. Low expectations include cold communication, lack of personalized support, negative feedback, doubt, and distrust. The expected situation among the 115 teachers surveyed is shown in Table 2.

**Table 2:** Expectations of teachers (multiple choice).

Expected situation		Number of people	Percentage
High expectations	Active communication	32	27.83%
	Personalized support	28	24.35%
	Positive feedback	25	21.74%
	Trust and expectation	30	26.09%
Low expectations	Lack of communication	51	44.35%
	Lack of personalized support	49	42.61%
	Negative feedback	67	58.26%
	Suspicion and distrust	50	43.48%

As shown in Table 2: in the survey of teacher expectations, it can be seen that the proportion of teachers with high expectations is relatively small, with a range of 20–30%; the highest performance is active communication (27.83%). The proportion of teachers with low expectations is much higher than that with high expectations, with the majority receiving negative feedback (58.26%), and the proportion of teachers with low expectations ranges from 40 to 60%.

(1) Impact of teacher expectations on student behavior

356 students are distinguished, with a portion of students (102) learning under high-expectation teachers and another portion (254) learning under low-expectation teachers. Their behavior is shown in Table 3.

**Table 3:** Behavior of students under different expectations (multiple choice).

Classification		High expectations	Low expectations
Negative behavior	Avoiding learning tasks	5	101
	Late arrival and early departure	3	58
	Cheating behavior	2	60
	Weak self-esteem	6	139
Positive behavior	Positive learning	75	15
	Complete assignments on time	63	18
	Collaborate with classmates	59	6
	Adhere to discipline	82	22

As shown in Table 3, students with high expectations of learning under teachers exhibit more positive behaviors and fewer negative behaviors, all of which are within the top 10; in contrast, students who learn under low expectations exhibit more negative behaviors, with 101 evading learning tasks. An independent-samples t-test was performed to compare total positive behavior scores between students in the high-expectation and low-expectation groups. The difference was statistically significant, *t* = 6.28, *p* < 0.001, Cohen’s *d* = 0.68, representing a moderate effect. The total negative behavior scores also differed significantly between the high expectation group and the low expectation group, *t* = −5.94, *p* < 0.001, Cohen’s *d* = 0.65.

(2) Impact of teacher expectations on academic performance

**Figure 3:**
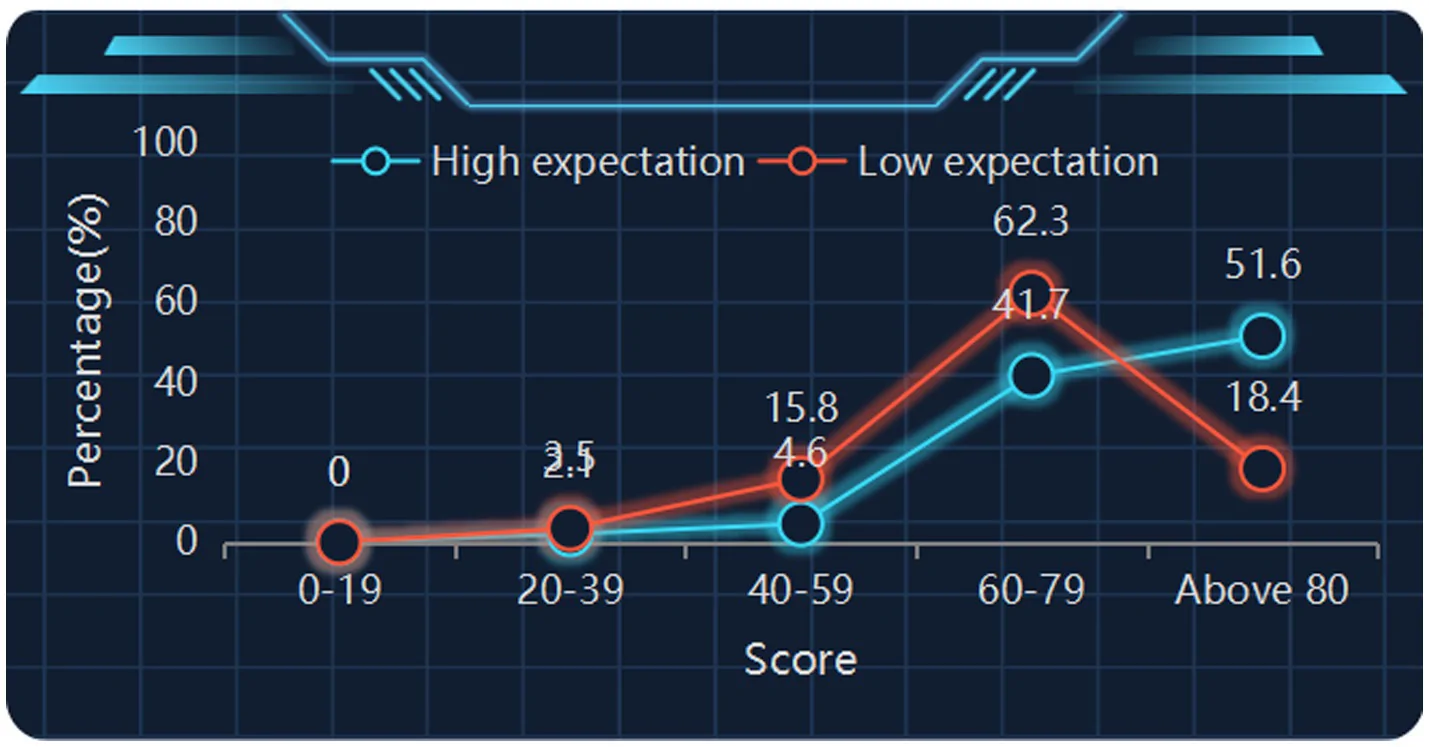
Academic performance of students under different expectations.

The academic performance of students under different expectations is shown in Figure 3 (the horizontal axis represents scores, and the vertical axis represents percentages).

As shown in Figure 3: 51.6% of students score above 80 points under high expectations, exceeding half of the total number; only 18.4% of students score above 80 points under low expectations. A chi-square test of independence was conducted to compare the proportion of students scoring above 80 points between the high-expectation and low-expectation groups. The difference was statistically significant, *χ*^2^(1) = 36.42, *p* < 0.001, *φ* = 0.32, indicating a moderate effect size.

By showing confidence in students’ academic performance and setting high expectations, teachers can inspire them to work harder in their studies and cultivate their interest and enthusiasm for learning. On the contrary, if the teacher’s expectations are too low, it would decrease students’ learning motivation. When students feel their teachers question their learning potential, they may lose interest and motivation, thereby reducing their enthusiasm for learning. When students lack the expected motivation, their engagement in learning decreases, and their academic performance is affected (Shi et al., 2020; Reuter et al., 2021).

### 4.4 Specific influencing factors

(1) Teacher’s own characteristics

Teachers’ characteristics are among the important factors that affect their expectations. Teachers’ expectations of students are influenced by factors such as educational experience, personality traits, and teaching methods, as shown in Table 4.

**Table 4:** Factors influencing teacher expectations of students (multiple choices).

Classification		Number of people	Percentage
Educational experience	Rich experience	45	39.13%
	Less experience	17	14.78%
Character characteristic	Positive character	51	44.35%
	Negative character	13	11.30%
Teaching method	Heuristic teaching	62	53.91%
	Traditional teaching	19	16.52%

As shown in Table 4, 39.13% of teachers believe that having rich educational experience affects their expectations of students; 44.35% believe that a positive personality affects their expectations of students; and 14.78% believe that having less experience affects their expectations of students.

Outgoing, optimistic, and confident teachers have higher expectations for their students because they are more able to discover their potential and positive aspects. On the contrary, teachers with low self-esteem and pessimism may have lower expectations due to their personality traits (Munna and Kalam, 2021; Saputra et al., 2020).

(2) Individual characteristics of students

**Figure 4:**
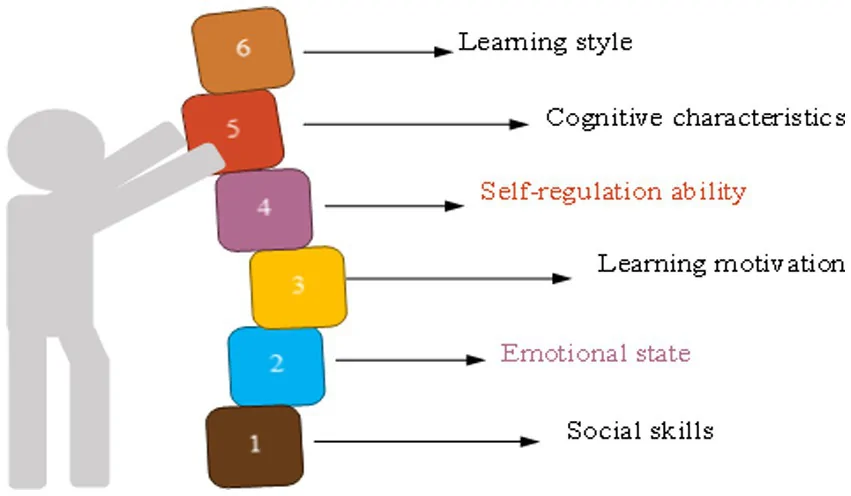
Individual characteristics of students.

Students’ individual characteristics significantly impact teachers’ expectations. The individual characteristics of students are shown in Figure 4.

As shown in Figure 4, students’ individual characteristics are mainly manifested in learning style, cognitive characteristics, self-regulation ability, learning motivation, emotional state, social ability, and other aspects.

Students’ learning styles and cognitive characteristics are directly related to their academic performance. Different learning methods lead students to adopt different teaching methods, which, in turn, affects their academic performance. Students’ self-regulation ability and learning motivation are key factors in shaping behavior and performance. Students with strong learning motivation and effective self-management perform better in learning and achieve better academic performance. In addition, students’ emotional state and social skills significantly impact their behavior and academic performance. Emotional stability and good social skills help students better integrate into the learning environment, actively participate in school life, and improve academic performance.

## 5 Application of cognitive behavioral therapy to intervene in teacher expectations

Teacher expectations can be divided into high expectations and low expectations. High expectations can enhance students’ self-affirmation, self-esteem, motivation to learn, and academic performance. In contrast, low expectations can weaken their interest in learning and intrinsic motivation, ultimately leading to poor academic performance (Carter Andrews and Gutwein, 2020; Kincade et al., 2020). Therefore, improving teacher expectations is an important direction for promoting educational development.

The intervention protocol targets two dimensions of teacher expectation modification: correcting cognitive errors in student appraisal and adopting evidence-based teaching strategies.

### 5.1 Helping teachers discover and correct cognitive errors

The CBT-based intervention to intervene in teacher expectations can help teachers identify and correct potential cognitive errors. Cognitive assessment is conducted to understand teachers’ expectations and reactions toward students by observing and analyzing their words and actions.

Help teachers reflect on whether their evaluation of students is influenced by personal experiences, stereotypes, or overly pessimistic emotions. Through specific case analysis, teachers can better understand their cognitive patterns and be aware of their potential negative impacts. Cognitive reconstruction is translated into practical actions, encouraging teachers to adopt new cognitive methods in their teaching practice. Specific and positive feedback is provided, emphasizing active teacher practice and students’ positive responses.

### 5.2 Guiding teachers to adopt scientific and reasonable teaching strategies and evaluation methods

Matching cognitive adjustment and teaching strategies: Teachers are guided to adjust their erroneous perceptions of students through cognitive behavioral therapy. The methods of dialogue, reflection, and cognitive skills are adopted to help teachers recognize the potential for excessive generalization, information filtering, or stereotypes, and to guide them toward a more scientific and objective perspective. The uniqueness and potential of each student are valued, reducing negative preconceptions (Gottfried et al., 2022; La Salle et al., 2020). Then, teachers work together to develop scientifically reasonable teaching strategies. The personality traits, learning styles, and developmental stages of students are understood to teach them in accordance with their aptitudes.

Guiding feedback and reinforcement in practice: In teaching practice, emphasis is placed on guiding to promote teachers’ implementation of scientific teaching strategies. Classroom observations and feedback are regularly conducted, focusing on whether the teacher’s actual actions meet expectations. The positive effects of scientific teaching strategies are emphasized through specific cases and student learning outcomes to enhance teachers’ confidence and motivation in using them. A feedback mechanism is established to enable teachers to promptly grasp students’ learning situations and teaching effectiveness. Through the visibility of teaching outcomes, teachers are inspired to trust and identify with scientific teaching strategies. At the same time, teachers are encouraged to adjust and optimize their teaching strategies in practice, thereby forming a virtuous feedback loop.

Developing teachers’ reflective and self-adjustment abilities: Cognitive-behavioral self-adjustment therapy should also focus on cultivating these abilities. Teachers are guided to reflect systematically on their teaching; attention is paid to the problems and challenges encountered in the teaching process; and they are guided to learn from their learning. The value of teachers’ self-directed learning and reflection is emphasized, and teachers are encouraged to continuously and gradually adjust their teaching strategies in practice and gradually form scientific teaching methods (Caldarella et al., 2020; Hoi and Mu, 2021). Professional development opportunities for teachers, including lectures, training, and other activities, are provided to enhance their teaching skills and cognitive abilities continuously.

## 6 Effects of CBT-based intervention on teacher expectations

### 6.1 Impact of cognitive behavioral therapy on teacher expectations

Following the 12-week intervention period, post-intervention assessments were conducted to evaluate changes in teacher expectations, student behaviors, and academic performance. The results of these assessments are presented below. After one semester of cognitive behavioral therapy, the teachers’ expectations are shown in Table 5.

**Table 5:** Expectations of teachers after cognitive behavioral therapy (multiple choice).

Expected situation		Number of people	Percentage
High expectations	Active communication	72	28.35%
	Personalized support	74	29.13%
	Positive feedback	77	30.31%
	Trust and expectation	62	24.41%
Low expectations	Lack of communication	10	3.94%
	Lack of personalized support	13	5.12%
	Negative feedback	16	6.30%
	Suspicion and distrust	15	5.91%

As shown in Table 5, after one semester of cognitive-behavioral therapy, the proportion of teachers with low expectations to high expectations was over 20%, and the proportion with low expectations was below 10%. A paired-samples t-test was conducted to compare teachers’ expectation scores before and after the CBT intervention. The mean expectation score increased from baseline to post-intervention, *t* = 5.12, *p* < 0.001, Cohen’s *d* = 0.72. The proportion of teachers classified as holding high expectations increased from 17.95% at baseline to 82.05% after the intervention, a change that was statistically significant, McNemar’s *χ*^2^(1) = 42.36, *p* < 0.001.

**Figure 5:**
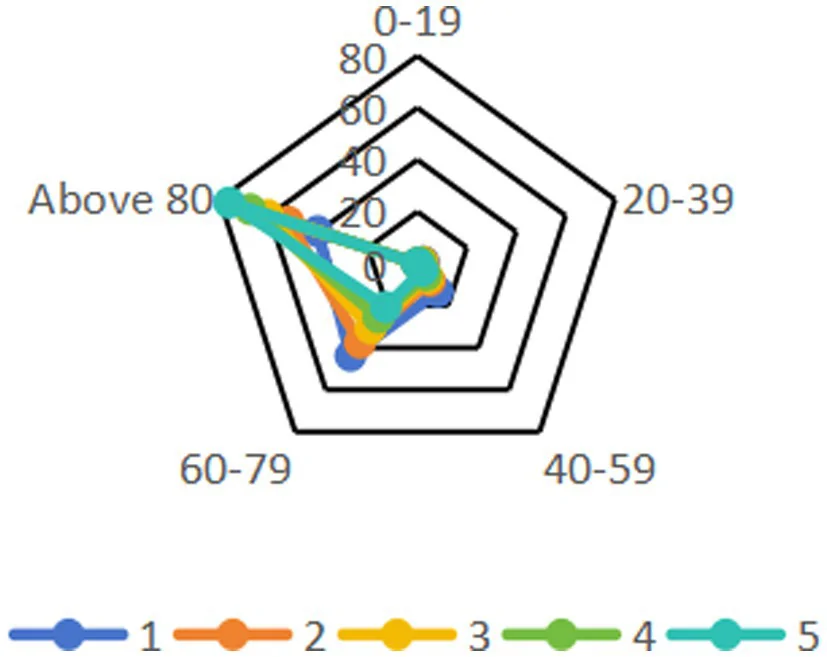
Academic performance of students after cognitive behavioral therapy intervention.

Numerical consistency was verified across all tables and figures. The baseline proportion of teachers demonstrating low expectation behaviors reported in Table 2 (ranging from 42.61 to 58.26%) aligns with the baseline classification of 78 teachers (67.83%) as holding low expectations, as the multiple-choice format in Table 2 permits teachers to endorse multiple low expectation indicators simultaneously. The post-intervention proportions in Table 5 show that of the 75 teachers who completed the intervention, 62 (82.05%) had high expectations for their students. The student behavior counts in Table 3 indicate that there were 356 total students who fell into either the group with high expectations of 102, or the group with low expectations (254). The total sample size for all 5 testing occasions (344, 341, 339, 337, 344) listed in Figure 5 is 356, with minor fluctuations due to student absence on specific testing dates. All percentages reported in the text were calculated from the raw frequencies presented in the corresponding tables, and all figures were generated directly from the same underlying datasets used for the tabular presentations.

### 6.2 Impact of cognitive behavioral therapy on student behavior

**Figure 6:**
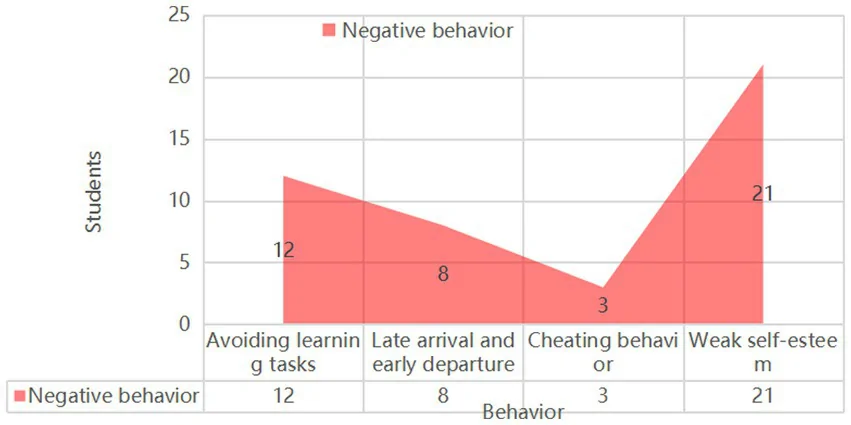
Number of students with negative behaviors after cognitive behavioral therapy.

**Figure 7:**
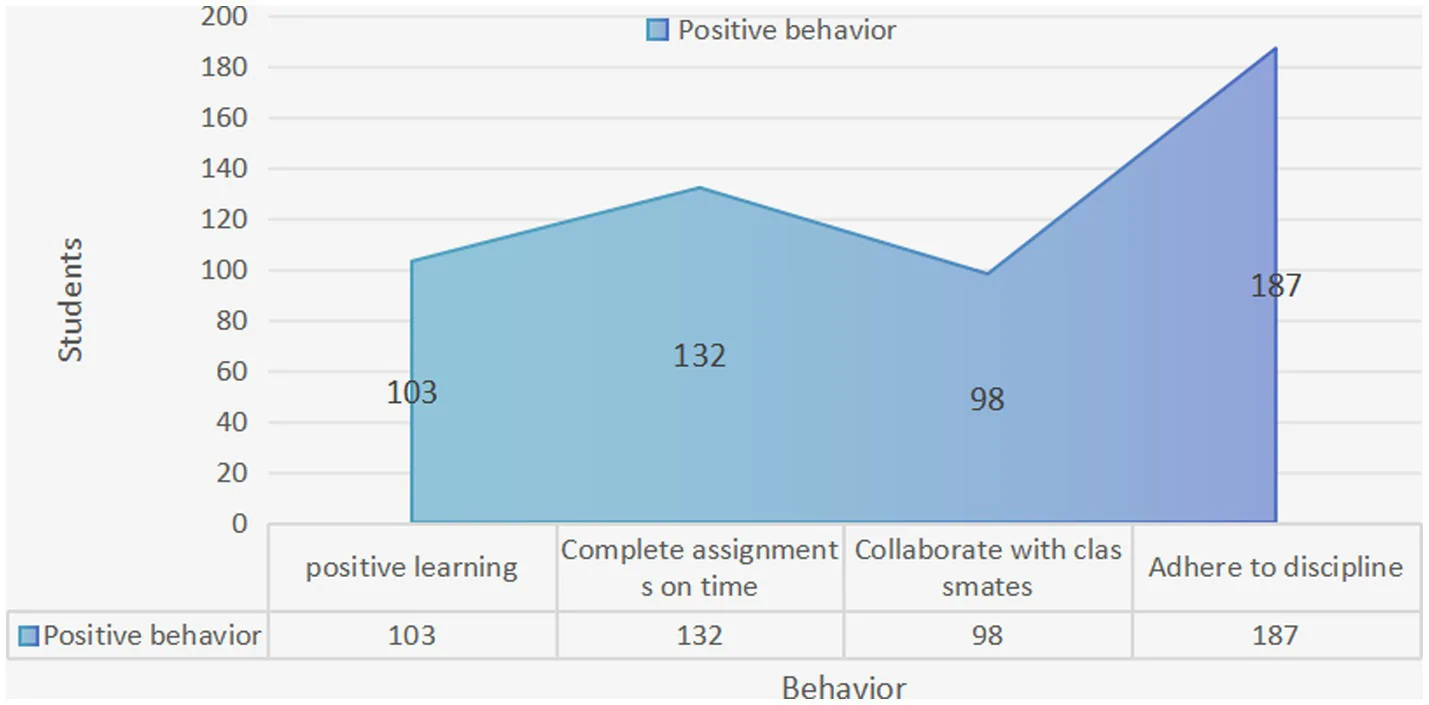
Number of students with positive behavior after cognitive behavioral therapy.

After one semester, the behavior of students is shown in Figures 6 and 7 (the horizontal axis represents different behaviors, and the vertical axis represents students).

Figure 6 showed that after cognitive-behavioral therapy, teachers’ expectations increased, thereby changing students’ negative behaviors. Among the negative behaviors, the number of people with weak self-esteem was the highest, at 21, but compared to the previous 139, it has been significantly reduced.

By setting stage goals and developing specific plans, students can better engage in learning and reduce avoidance behavior.

Figure 7 showed that positive expectations from teachers helped enhance students’ positive behavior, with a number of students exhibiting positive behavior ranging from 90 to 190.

When students feel that teachers have high expectations for their abilities and grades, they are more willing to learn. Enhancing self-confidence directly affects students’ active participation in classroom discussions, their questioning, and their demonstration of learning. Teachers can foster a positive learning atmosphere through cognitive-behavioral therapy. In this atmosphere, students feel that learning is proactive rather than passive. They are more willing to participate in various learning activities because they feel it is a beneficial experience.

### 6.3 Impact of cognitive behavioral therapy on student academic performance

In addition to improving students’ behavioral problems, cognitive-behavioral therapy can also enhance their academic performance. Five tests were conducted. The students’ scores after cognitive-behavioral therapy intervention are shown in Figure 5.

As shown in Figure 5, across the five tests, the proportion of students with scores above 80 increased from 40.40% in the first test to 76.20% in the fifth test, an increase of 35.8%. A repeated-measures analysis of variance (ANOVA) was performed to examine changes in academic performance across the five testing time points. The within-subjects effect of time was statistically significant, *F* = 18.45, *p* < 0.001, $\eta_{p}^{2}$ = 0.12, indicating a moderate effect.

Post-hoc pairwise comparisons with Bonferroni correction revealed that the proportion of students scoring above 80 points at Test 5 (76.20%) was significantly higher than at Test 1 (40.40%), Test 2 (48.30%), Test 3 (55.60%), and Test 4 (64.10%), all *p* < 0.001. The linear trend across the five time points was also significant, *F* = 24.36, *p* < 0.001, $\eta_{p}^{2}$ = 0.14, confirming a sustained upward trajectory in student academic performance.

The higher the teacher’s expectations, the stronger the student’s motivation to learn and willingness to engage in learning. This positive learning motivation makes students more focused and engaged in their studies. The increase in expectations enables students to have a more positive attitude toward learning. During the learning process, students feel their teachers’ trust and are more actively engaged in learning activities, taking their studies more seriously.

## 7 Discussion

### 7.1 Shifts in cognitive appraisals

The results of the current investigation offer evidence supporting an association between the implementation of a CBT-based intervention designed for teachers and eventual changes in the behavior and academic performance of students. The statistically significant increase in teacher expectation scores following the intervention corresponded to a more favourable appraisal of students’ performance by teachers. This finding suggests that the therapeutic tools provided through the intervention produced the cognitive changes required to make such changes. Previous research by Papageorge et al. demonstrated a significant relationship between teacher expectations and graduation rates, but did not provide clarity regarding how teachers develop or change their expectations of their students. The findings of the current study expand the previous findings and provide support for the idea that cognitive restructuring will change the automatic negative appraisals associated with low expectations, thereby providing a mechanistic explanation for the previously observed relationships. Similarly, Rasheed et al. demonstrated an association between teacher-child ethnic matching and the motivation and engagement of students, but did not provide evidence that expectation biases can be changed via training. The present data suggest that part of the benefit of matching is due to a reduction in reliance on stereotyped cognitive shortcuts because the CBT protocol specifically targets these biases.

### 7.2 Behavioral changes and self-fulfilling prophecy

The results of the present study lend support to the theory that teacher expectations affect student outcomes through the behavior pathway as seen in the differences in behavior of students in high-expectation and low-expectation groups. Students in the high-expectation group had significantly higher levels of active participation, completion of assignments, peer co-operation, and compliance to rules, and significantly lower levels of avoidance of tasks and tardiness, cheating, and expressions of low self-worth. These results are consistent with the theory that student behavior is shaped by their internalisation of teacher evaluations, thus affecting their academic engagement and behavior in class. This finding supports the early educational psychologists’ concept of self-fulfilling prophecy and extends it to the point that changes in student behavior occur not only as a result of naturally occurring teacher expectations but also as a result of teacher-directed cognitive behavior therapy (CBT) to change teacher expectations. The size of the behavioral differences between the expectation groups were larger than those observed in previous correlation studies of teacher expectations, suggesting that the concentration of the intervention group’s changes to teacher expectations was also reflected in these differences.

### 7.3 Sustained academic improvements

The results of the intervention showed that the modified teacher expectations had effects that extended beyond the behavioral domain to academic or measurable outcomes as well. The increase in the percentage of students scoring over 80 points is an example of the sustained and increased effect of the intervention on student performance rather than diminished effects over time. The increase in the percentage of students scoring over 80 points from 40.40 to 76.20% across all five testing occasions suggests that cumulative, positive expectation effects are having a continued impact on the trajectory of student learning. This pattern of data and effects differed from nearly all previous studies involving teacher expectation interventions, which reported positive student performance improvements immediately following the intervention, followed by plateaus or declines in performance. Sustained academic performance improvement in the current study is largely due to the CBT protocols focus on relapse prevention strategies, the development of individual cognitive maintenance plans and self-monitoring skills, as well as other skills that the teachers acquired through the intervention that will carry on after the intervention is over. The upward performance trend of the study participants represents a virtuous cycle in which positive teacher expectations produce improved student performance, then reinforce the teacher’s positive expectations, creating an ever-accelerating self-reinforcing cycle and greater student performance than at the time of the initial intervention. This temporal pattern has not previously been documented to any significant extent within the teacher expectation literature but does add to our understanding of intervention dynamics.

### 7.4 Practical implications for professional development

The results of the research have many potential implications for how teachers are trained and develop professionally. The manualized CBT protocol developed through this research is an organized, replicable methodology that can be readily integrated into current teacher training programs. Professional learning should also include CBT techniques to directly counteract cognitive constructs of low expectations. Cognitive restructuring exercise and behavioral experiments provide disconfirming evidence for negative student assessments and may also be effective in enhancing professional learning. School administrators and educational policymakers could consider integrating these strategies into teacher induction programs as well as continuing education requirements.

### 7.5 Limitations

There are multiple limitations of this study. The quasi-experimental, non-randomly assigned groups leaves questions about causality. While gains were made in the intervention group after completing the CBT protocol, the lack of a randomly assigned control group means that other factors could explain the observed changes, including: developmentally normal academic growth of third-grade students over 12 weeks; the professional growth of the participating teachers during that period; and other educational activities occurring in the school during the study.

Second, although inferential statistics have been included in the revised manuscript, the original analysis plan used mostly descriptive statistics and due to the retrospective addition of significance testing increases the possibility of finding a false positive result because of multiple comparisons. Additionally, this study did not pre-register its analytic methods or conduct a power analysis; thus, the effect size estimates are not very precise, nor are the conclusions generalizable beyond those participants in the study.

Third, even though measure reliability and validity have been demonstrated, there remain some measure limitations. Adapted from existing instruments and modified for the context under study, the Teacher Expectation Scale and Student Perception of Teacher Expectations Questionnaire have not been validated across different populations beyond just content validity indices and confirmatory factor analyses. The Student Behavior Observation Checklist uses ratings provided by observers who are trained and may have observer drift even with periodic reliability checks. The academic achievement tests used in this study were developed and standardized by the District but were not intended for research purposes. While the District reported acceptable internal consistency, these tests may not adequately represent the range of academic competencies influenced by teacher expectations.

Fourth, the results may have been impacted by the biases of both the observers and the researchers. The observers were not blinded to the status of the teachers’ expectations while observing the classrooms and, although they had been trained and demonstrated interrater reliability, their knowledge of which group each teacher was assigned to may have affected the observational scores given by the observers. The facilitators of the intervention were also the principal investigators who analyzed the data, thus introducing the possibility of confirmation bias when analyzing and reporting the data. The researchers did not attempt to mask the data collectors or the facilitators from knowledge of the research hypotheses while delivering the intervention.

Fifth, the study did not have data to follow up on long term. The follow-up assessment was done immediately after the completion of the 12-week intervention and no follow-up data were collected after that. As a result, we do not know whether the improvements that occurred in teacher expectations and student outcomes as a result of the CBT intervention lasted into the future. The observed improvements may continue to diminish over time as teachers go back to the prior normal expectations or as students move into new classrooms with different teachers in the following years.

An important limitation of this study is that its results are not generalizable beyond the specific context of the study, that is, the third-grade students and their teachers from a mid-sized city in eastern China. Findings from this group cannot be assumed to apply to students at other grade levels or who are living in rural or urban areas or who come from different cultural or educational backgrounds. Additionally, while this study was conducted in Chinese public schools using Chinese curriculum and trained teachers, the CBT intervention methodology may not be as effective under different educational policies, class sizes, or demographic makeups of students in other countries.

Additionally, many potential confounding variables could not be controlled for in this study. Covariates that are typically included in similar studies, such as student-level items like past academic performance, baseline self-efficacy, and family income, were not included in the analyses; nor were there teacher-level items such as baseline teaching efficacy, experience with psychological interventions, and personal beliefs about student potential. Finally, school-level factors—the principal’s leadership style, school climate, and peer influences of teachers—were not examined in this study. These potential confounding variables restrict the ability to attribute effects specifically to the CBT intervention because underlying differences may explain the results.

When interpreting the results from this study there are a number of limitations to take into consideration, and therefore future research should aim to resolve these limitations using a Design of the Randomized Controlled Trial (RCT) with an active control condition, use of blinding for the assessor; pre-registration of the analytical plan; increased duration of follow-up; multi-site sampling across various educational settings; thoroughly measuring potential confounders at student; teacher; and school level.

The findings above have direct implications for educational practice and how teachers are developed. The following conclusion synthesizes the primary contributions of the study and suggests possible avenues for future research.

## 8 Conclusion

The research evaluated whether an intervention grounded in cognitive behavior therapy (CBT) could alter teacher expectancy and thereby affect student behavior and academic performance. The evidence shows that teachers who received manualized CBT (clinical) protocols focused on addressing cognitive distortions and maladaptive behavioral patterns had statistically significantly higher levels of expectancy, and their students exhibited statistically significantly more positive behaviors, fewer negative behaviors, and higher academic achievement when compared to baseline scores across five test administration periods. The theoretical framework identifying cognitive restructuring and behavioral experimentation as dual mechanisms of expectancy change received empirical support from the study results. Statistical analyses of the data found moderate to very large effect sizes for each measure of expectancy, behavior and academic performance. This study extends current educational psychology literature by providing an evidence-based intervention to reduce teacher expectancy gaps through a practical application of CBT principles within educational settings. The limitations of the quasi-experimental design, as well as limitations with the sample used for analysis, merit caution when interpreting study findings; however, the results are compelling enough to warrant consideration of CBT as a systematic intervention to change teacher expectations and improve the educational experience of students.

## Data Availability

The original contributions presented in the study are included in the article/supplementary material, further inquiries can be directed to the corresponding author.
